# Analytical sensitivity of a method is critical in detection of low-level BRCA1 constitutional epimutation

**DOI:** 10.1038/s41598-023-43276-7

**Published:** 2023-09-26

**Authors:** Filip Machaj, Katarzyna Ewa Sokolowska, Konrad Borowski, Szymon Retfiński, Dominik Strapagiel, Marta Sobalska-Kwapis, Tomasz Huzarski, Jan Lubiński, Tomasz Kazimierz Wojdacz

**Affiliations:** 1 Independent Clinical Epigenetics Laboratory, Pomeranian Medical University in Szczecin, Unii Lubelskiej 1, 71‐252 Szczecin, Poland; 2 Biobank Laboratory, Department of Oncobiology and Epigenetics, Faculty of Biology and Environmental Protection, University of Lodz, Pomorska 139, 90‐237 Lodz, Poland; 3 Department of Genetics and Pathology, Pomeranian Medical University in Szczecin, Unii Lubelskiej 1, 71‐252 Szczecin, Poland

**Keywords:** Epigenetics analysis, Breast cancer

## Abstract

Recent reports based on a substantial number of cases, warrant need for population-based research to determine implications of constitutional methylation of tumor suppressor genes such as *BRCA1* occurring in healthy tissue in the prediction of cancer. However, the detection of the constitutional methylation in DNA extracted from blood has already been shown to be technologically challenging, mainly because epimutations appear to be present in blood at a very low level. The analytical sensitivity required for low-level methylation detection can be provided by NGS, but this technique is still labor and cost-intensive. We assessed if PCR-based MS-HRM and BeadChip microarray technologies, which are standardized and cost-effective technologies for methylation changes screening, provide a sufficient level of analytical sensitivity for constitutional *BRCA1* methylation detection in blood samples. The study included whole blood samples from 67 healthy women, 35 with previously confirmed and 32 with no detectable *BRCA1* promoter methylation for which we performed both MS-HRM based *BRCA1* gene methylation screening and genome wide methylation profiling with EPIC microarray. Our results shown, that low-level *BRCA1* methylation can be effectively detected in DNA extracted from blood by PCR-based MS-HRM. At the same time, EPIC microarray does not provide conclusive results to unambiguously determine the presence of *BRCA1* constitutional methylation in MS-HRM epimutation positive samples. The analytical sensitivity of MS-HRM is sufficient to detect low level *BRCA1* constitutional epimutation in DNA extracted from blood and BeadChip technology-based microarrays appear not to provide that level of analytical sensitivity.

## Introduction

Breast cancer continues to be the most common neoplasm in females and recent reports suggest its rising incidence, especially in young women^1^. Germline mutations in *BRCA1* are the most researched genetic lesion that increase the risk of breast cancer, but are present in only up to 5% of total breast cancer cases in Poland^2^. Constitutional methylation of *BRCA1* detectable in blood cells was first associated with an increased risk of breast cancer tumors with features resembling BRCA1-mutated tumors over a decade ago^3^. Since that publication, several other studies investigated the prevalence of the methylation of *BRCA1* promoter in peripheral blood and its association with cancer risk, but the results of those studies were frequently discrepant. The inconsistency of the results of previous studies can most likely be attributed to limited statistical power and different methylation detection methods used^3–6^. Especially, that the constitutional methylation of *BRCA1* has been shown to be present at very low level in DNA extracted from whole blood. Thus, the sensitivity of the method for methylation detection appears to be critical in studies of constitutional *BRCA1* gene methylation phenomenon. A recent study added a significant body of evidence for the utility of *BRCA1* constitutional epimutation testing in blood in the prediction of both ovarian and breast cancers^7^. The study included 2478 of a triple-negative breast cancers (TNBCs) and 3493 high-grade serous ovarian cancers (HGSOCs) cases and controls without a germline pathogenic variant of *BRCA1* and concluded that testing for *BRCA1* epimutation in blood may have implications in cancer prediction. This study also used very sensitive technology for mutation changes detection (Next Generation Sequencing—NGS), and again detected methylation levels of *BRCA1* in blood were low. NGS can provide required sensitivity for low level methylation detection but is still not cost-effective in the context of sequencing single PCR products. Additionally, the technique is laborious, especially because NGS data still require bioinformatics expertise to analyze. Thus, standardized, highly sensitive, and cost-effective methods for methylation detection are needed if *BRCA1* methylation testing of large populations is considered.

The most frequently used methods for locus specific methylation changes testing are PCR-based methods and the most cost-effective method for genome-wide methylation studies are Methylation BeadChip microarrays (Illumina Inc). The PCR-based methods can provide state-of-the-art sensitivity but there is no consensus whether BeadChip microarrays provide sensitivity sufficient to assess constitutional methylation changes.

The largest study investigating association of the genome-wide methylation changes in pre-diagnostic blood samples with breast cancer risk (three independent cohorts of matched cases and controls: EPIC; n = 162, NOWAC; n = 168, BGS; n = 548) based on BeadChip microarray technology, did not find methylation changes at *BRCA1* gene^8^, similarly to study by Severi G. et al.,^9^. At the same time a study based on blood derived from 30 breast cancer cases with BRCA1-like phenotype that also used BeadChip microarray, found elevated methylation levels in seven cancer cases with BRCA1-like phenotype, at the CpG sites within *BRCA1* promoter that are most studied for constitutional methylation changes but reported methylation differences were less than 10%^10^. A more recent study found methylation differences at only one of the CpG sites in the *BRCA1* promoter in the blood of breast cancer women and pre-diagnostic breast cancer cases and again here detected methylation level difference were less than 2%^11^.

With the new generation of the BeadChip microarrays dominating populational methylation studies, we performed a head-to-head comparison of analytical sensitivity of PCR-based Methylation Sensitive High-Resolution Melting (MS-HRM) and MethylationEPIC microarray for the *BRCA1* constitutional methylation detection in DNA extracted from whole blood.

## Methods

Our study included 67 blood samples from healthy women, 35 cases from our previous study tested positive for *BRCA1* constitutional methylation^12^, and 32 controls with no detectable *BRCA1* promoter methylation. The women underwent *BRCA1/2* germline mutation screening at the International Hereditary Cancer Center in Szczecin in the years 2008–2015 with no germline mutations detected^13^. The mean age of the women positive for *BRCA1* epimutation at sampling was 60.75 years (37–90) and for controls was 57.68 (50–69). The DNA was extracted using the salting-out method and bisulfite conversion of 300ng DNA was performed using EZ DNA Methylation Gold Kit (Zymo Research, Irvine, CA) following manufacturer protocol. The epimutation testing was performed using EpiMelt BRCA1 MS-HRM methylation screening kit (MethylDetect ApS, Denmark) and LightCycler® 480 High-Resolution Melting Master (Roche, Germany) using Q – qPCR Instrument (QuantaBio, UK). The kit used for methylation testing included apart from methylated and non-methylated controls, an assay calibration control that controls for 1% sensitivity of the methylation detection. The theoretical (not accounting for degradation of the template during bisulfite conversion and losses during sample preparation) concentration of the template in PCR was 5.45 ng/ul. The PCR was performed in triplicates in 50 cycles. Each cycle consisted of denaturation at 95 °C for 15s, primer annealing at 59 °C for 15s (as recommended by the manufacturer), and extension at 72 °C for 15s. The melting included an initial denaturing step at 95 °C for 15s and a stepwise increase in temperature from 67 to 90 °C with a rate of 0.025 °C/s. Then, the data were analyzed with Q-qPCR v1.0.2 software provided with the Q—qPCR Instrument. The Illumina MethylationEPIC v1.0 BeadChip (EPIC, Illumina Inc.) data were processed with the ChAMP package^14^ including data normalization with the BMIQ method. In total, 733,236 probes passed QC. No statistically significant differences between analyzed samples were detected with the EpiDISH R package modified as described in^15^ and none of the processed microarrays was a technical outlier according to MethylAid package^16^. The R version 4.1.3 was used for data processing and statistics calculation. All methods were performed in accordance with the relevant guidelines and regulations.

### Ethics approval and consent to participate

The study was approved by the Ethics Committee of the Pomeranian Medical University in Szczecin. Each patient has signed an informed consent for study participation.

## Results

### *BRCA1* methylation levels in whole blood samples detected with MS-HRM are low

There is some discrepancy between studies regarding the part of the *BRCA1* gene promoter that is targeted by assays aiming to detect constitutional methylation. The initial publication that demonstrated the association of the low-level promoter methylation of *BRCA1* in blood with the development of BRCA1-like breast cancer (with tumor displaying features typical for cancer with mutated *BRCA1* gene)^3^, targeted the region in which methylation changes had previously been associated with decreased *BRCA1* mRNA levels in clinical breast cancer specimens^17^. The same region was targeted in subsequent studies that confirmed initial findings^4^ as well as our previous study of *BRCA1* constitutional methylation in Polish population^12^. The MS-HRM assay we used in this study targets five CpG sites (chr17: 41277381–41277382, chr17: 41277389–41277390, chr17: 41277392–41277393, chr17: 41277394–41277395, chr17: 41277400–41277401) in that region.

**Figure 1:**
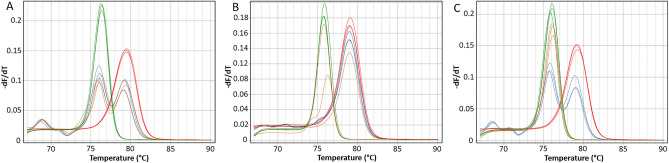
Examples of the MS-HRM results for the BRCA1 constitutional methylation positive and negative samples: (**A**) sample positive for methylation run at 59 °C; (**B**) sample positive for methylation but not reproducibly detectable run at 61 °C; (**C**) sample negative for methylation run at 59℃. Color legend: red—100% methylated control, green—0% non-methylated control, blue—1% calibration control, orange—test sample.

We reproducibly detected methylation in 29 samples in that region (Fig. 1A). In six of the screened samples, we did detect methylation, however not in all technical PCR replicates (Fig. 1B), and no methylation was detected in any of the control samples (Fig. 1C). The advantage of the MS-HRM protocol is that the sensitivity of the technology is annealing temperature dependent^18^. Therefore, in the attempt to reproducibly detect methylation in six samples in which the methylation was not detected in all PCR replicates, we re-tested those samples at raised the annealing temperature of 62 °C, and additionally doubled the amount of the template in the PCR. Nevertheless, this modification did not increase the reproducibility of the detection of methylation in those cases (data not shown) and the methylation detection was again not consistent between PCR replicates. Those results again indicates that analytical sensitivity of the method is critical for *BRCA1* epimutation detection in blood as this epimutation may be present a very low level.

### Standard data processing of EPIC microarray data does not detect *BRCA1* gene methylation changes

The EPIC microarray targets 59 CpG sites within the *BRCA1* gene. We used the ChAMP pipeline (ChAMP.DMP) with default settings to identify differently methylated probes in the *BRCA1* gene and data from 41 probes retained in the analysis after standard data quality control. The methylation levels at six of those probes were statistically significantly (FDR corrected p-value < 0.05) different between cases and controls in our study (Supplementary Data 1). However, the CpG sites targeted by those probes appear to be randomly distributed throughout *BRCA1* 1500TSS region. Moreover, none of those CpG sites mapped to, or was in the proximity to the region where constitutional methylation of *BRCA1* was detected.

### The analytical sensitivity of the assay is essential for the *BRCA1* constitutional methylation detection in blood

**Figure 2:**
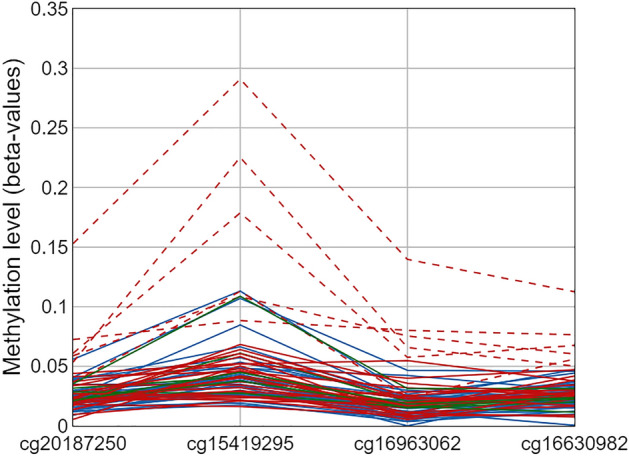
Methylation levels at four CpG sites within the region targeted by MS-HRM assay with samples positive for methylation indicated with red dashed and solid lines, samples negative for methylation indicated with blue lines, and samples positive for methylation but not reproducibly indicated with green lines.

Four of the CpG sites targeted by the MS-HRM assay we used here, are also targeted by the EPIC BeadChip microarray. To assess the sensitivity of the MS-HRM and microarray we subdivided the MS-HRM results into three categories: positive methylation status (n = 29), methylation status detected stochastically (n = 6), and negative methylation status (n = 32) and compared methylation levels measured with MS-HRM for each of those groups with the methylation levels detected by EPIC microarray. Only six out of 29 samples positive for methylation in MS-HRM experiments (Fig. 2, samples indicated with red dashed lines) displayed methylation levels higher than the majority of the screened samples in microarray data. Apart from those samples, the methylation levels in samples positive for methylation (red solid lines Fig. 2), samples negative for methylation (blue solid lines Fig. 2), and samples positive for methylation but not reproducibly detectable (green solid lines Fig. 2) were very similar and around 5%, with only three samples exceeding that range for one of the screened probes.

We also tested mean methylation levels at the four probes targeted by MS-HRM for statistically significant differences between three groups of the samples divided according to MS-HRM results. The methylation level differences between compared groups were not statistically significantly different (Wilcoxon signed-rank test) and the largest observed mean methylation level difference was 2,4% at cg15419295, between samples positive for methylation and samples positive for methylation but not reproducibly detectable (Table 1).

**Table 1:** Comparison of mean methylation levels from EPIC microarray at four CpGs from the region targeted by MS-HRM assay between samples positive for methylation in MS-HRM analysis (denoted as Group 1), samples negative for methylation (denoted as Group 2) and samples positive for methylation but not reproducibly detectable (denoted as Group 3).

CpG	Group 1	Group 2	Group 3	Δ mean beta 1 versus 2	Δ mean beta 1 versus 3	Δ mean beta 2 versus 3
cg20187250	0.034	0.026	0.024	0.007	0.010	0.002
cg15419295	0.066	0.053	0.042	0.013	0.024	0.011
cg16963062	0.031	0.020	0.016	0.012	0.015	0.003
cg16630982	0.036	0.023	0.025	0.013	0.011	-0.002

## Discussion

A recent case–control study performed on thousands of cases and controls showed that constitutional methylation of *BRCA1* gene promoter, detectable in blood cells is significantly associated with the risk of incident triple-negative breast cancers (TNBCs) and high-grade serous ovarian cancers (HGSOCs)^7^. The results of this study are generally in line with the results of previous studies, (including ours^12^), but those previous studies included significantly smaller number of patients^3–6^.

Overall, there seems to be sufficient research evidence to consider the potential implications of the *BRCA1* constitutional methylation testing in normal tissue as well as other tumor suppressor genes for the prediction of cancer. However, the majority of studies reporting the association of the *BRCA1* constitutional epimutation with cancer risk also show that if constitutional methylation is present in blood samples, it is present at a very low level^4,19–21^. Our results confirm that especially that in a few samples in our study, the concentration of the template originating from methylated *BRCA1* promoter was so low that we were not able to reproducibly detect this epimutation in all PCR replicates.

The detection of low-level methylation is challenging from the technological point of view and requires highly sensitive methods. The use of the next generation sequencing, as in the most recent study overcomes the issue of method sensitivity. However, this technology is labor and cost-intensive. The PCR-based methods for methylation detection, as well as BeadChip technology-based microarrays, are significantly more cost and labor effective but detect methylation with varying sensitivity.

We compared the sensitivity of detection of methylation at the *BRCA1* locus between PCR-based MS-HRM and EPIC microarray and showed that the sensitivity of the microarray is not sufficient to unambiguously detect low levels of methylation. This is an important observation because the largest population-based studies investigating genome-wide methylation changes in the context of breast cancer predisposition using BeadArray technology do not report *BRCA1* methylation^8,9^ and our results clearly show that this may be attributed to the sensitivity of the method used in those studies.

In conclusion, an increasing number of studies report the association of methylation in healthy tissue with cancer incidence of not only *BRCA1* but also other genes with known roles in carcinogenesis^22^. Those studies warrant further research to determine if constitutional methylation of tumor suppressor genes is a cancer risk factor. However, with the results we report here the choice of the methylation detection method appears to be critical in future studies of constitutional methylation in healthy tissues. Only methods with high and quantifiable sensitivity should be used in those studies. Those findings also apply in the context of screening for *BRCA1* somatic epimutation in tumor tissue, when for example assessing the suitability of PARP inhibitors or platinum-based therapy for specific cancer patient groups.

### Supplementary Information

Supplementary Information.

## Data Availability

The raw genome-wide profiling methylation data are available from the corresponding author upon justified request.

## Abbreviations

BRCA1: BReast CAncer gene 1
TNBC: Triple-negative breast cancers
HGSOC: High-grade serous ovarian cancers
PCR: Polimerase Chain Reaction
MS-HRM: Methylation Sensitive High Resolution Melting
HRM: High Resolution Melting
