# Publisher Correction: Analytical sensitivity of a method is critical in detection of low-level BRCA1 constitutional epimutation

**DOI:** 10.1038/s41598-023-44744-w

**Published:** 2023-10-18

**Authors:** Filip Machaj, Katarzyna Ewa Sokolowska, Konrad Borowski, Szymon Retfiński, Dominik Strapagiel, Marta Sobalska-Kwapis, Tomasz Huzarski, Jan Lubiński, Tomasz Kazimierz Wojdacz

**Affiliations:** 1 Independent Clinical Epigenetics Laboratory, Pomeranian Medical University in Szczecin, Unii Lubelskiej 1, 71‐252 Szczecin, Poland; 2 Biobank Laboratory, Department of Oncobiology and Epigenetics, Faculty of Biology and Environmental Protection, University of Lodz, Pomorska 139, 90‐237 Lodz, Poland; 3 Department of Genetics and Pathology, Pomeranian Medical University in Szczecin, Unii Lubelskiej 1, 71‐252 Szczecin, Poland

Correction to: *Scientific Reports* 10.1038/s41598-023-43276-7, published online 26 September 2023

In the original version of this Article Filip Machaj was incorrectly listed as a corresponding author. The correct corresponding author for this Article is Tomasz Kazimierz Wojdacz. Correspondence and request for materials should be addressed to tomasz.wojdacz@pum.edu.pl.

The original Article has been corrected.

